# Collectanea Medica: Consisting of Anecdotes, Facts, Extracts, Illustrations, &c.

**Published:** 1824-06

**Authors:** 


### COLLECTANEA MEDICA:

CONSISTING OF

### ANECDOTES, FACTS, EXTRACTS, ILLUSTRATIONS, &c.

*Relating to the History or the Art of Medicine, and the Collateral Sciences.*

---

Floriferis, ut apes, in saltibus omnia libant,
Omnia nos, itidem, depascimur aurea dicta.

---

ART. I.—*Extracts from "Medical Jurisprudence."* By J. A. PARIS, M.D. &c. and J. S. M. FONBLANQUE, Esq.

*Suffocation* (continued).

II. *By Hanging.*—THE suspension of a person by means of a cord, or some other ligature, round the neck, by which death is produced by closing the trachea, and preventing respiration.

Although we are in this case bound to admit that the immediate cause of death is suffocation, yet we cannot deny that other injuries are often produced by hanging, such as

1. Pressure on the vessels.
2. Pressure on the nerves.
3. Fracture of the spine, and dislocation of the odontoid process.

1. *Pressure on the vessels.*—The red and livid hue of the face of persons killed by hanging, very naturally induced a belief that apoplexy was the immediate cause of death; while it is evident that the pressure on the jugular veins must necessarily so prevent the return of blood to the heart, as to produce an accumulation in the vessels of the brain. Dr. Hooper has a preparation of the brain of an executed criminal, in which blood is seen extravasated among the membranes; and various other cases have occurred, where dissection has clearly demonstrated the existence of those vascular congestions and sanguineous effusions, upon which apoplexy is supposed to depend. But this merely goes to prove that apoplexy occasionally takes place from hanging; it does not establish the fact of its being the common cause of death on such occasions. Gregory made the following experiment to show that it is to the interception of air that death is to be attributed: after having opened the trachea of a dog, he passed a slip-knot round the neck above the wound; the animal, though hanged, continued to live and respire; the air was alternately admitted, and easily expelled, through the small opening; but, as soon as the constriction was made below the orifice, the animal perished. Mr. Brodie hanged a dog, and, as soon as it became insensible, the trachea was opened below the ligature; upon which he breathed, and his sensibility returned.

2. *Pressure on the nerves of the neck.*—Although the pressure of a ligature on the nerves of the neck cannot be considered as the immediate cause of death in hanging, yet Mr. Brodie has very justly observed, that, if the animal recovers of the direct consequence of the strangulation, he may probably suffer from the effects of the ligature upon the nerves afterwards. Mr. Brodie passed a ligature under the trachea of a Guinea-pig, and tied it tight on the back of the neck with a knot; the animal was uneasy, but nevertheless breathed and moved about: at the end of fifteen minutes the ligature was removed; on the following morning, however, the animal was found dead. On dissection, no preternatural appearances were discovered in the brain, but the lungs were dark and turgid with blood, and presented an appearance similar to that which is observed after the division of the nerves of the eighth pair. I do not, observes Mr. Brodie, (*manuscript notes,*) positively conclude from this experiment that the animal died from an injury inflicted upon the nerves of the eighth pair, but I think that such a conclusion is highly probable; and it becomes an object of inquiry whether a patient, having recovered from hanging, may not, in some instances, die afterwards from the injury of the par vagum.

3. *Fracture of the spine, and dislocation of the neck.*—The death of a hanged person may occasionally take place by the luxation of the cervical vertebræ, and the consequent injury of the spinal marrow: this effect will be more likely to happen in heavy persons, and where the culprit suffers on a drop that precipitates him from a considerable height. It is said that Louis discovered that, of the two executioners in Paris and Lyons, one dispatched the criminal condemned to be hanged by luxating the head on the neck, whilst those who perished by the hands of the other were completely strangled.

An animal, when first suspended, is observed to make frequent, but ineffectual, attempts to inspire; violent convulsions of the whole body then ensue, but which are not to be considered as the indications of suffering, for they arise in consequence of the dark-coloured blood having reached the brain and spinal marrow, and the animal at this period is necessarily insensible. Hanging does not occasion a painful death.

The lips, nose, and all those parts in which the hue of the blood can be observed, exhibit a dark colour; the countenance is distorted, the eyes protruded, and frequently suffused with blood; the tongue is also forced out of the mouth, and frequently wounded, although it has been observed that this phenomenon will entirely depend upon the position of the rope; for that, when it presses above the thyroid gland, the tongue will be pushed back, in consequence of a compression upon the os hyoides; whereas, if the pressure be applied under the cricoid cartilage, it will have the effect of thrusting out the tongue. Blood is sometimes discharged from the ears. It is not unusual for the sufferer to void his urine, fæces, and even semen, in *articulo mortis*. The fingers are usually bent, the nails blue, and the hands nearly closed; and the whole physiognomy exhibits a highly characteristic appearance.

The dissection of a hanged person exhibits the same phenomenon as those described under the history of drowning, with the exception of the absence of water in the bronchiæ. With respect to the quantity of air found in the lungs, much discrepancy of opinion has existed. Dr. Goodwyn, in his experiments on respiration, found that the lungs of a person, who had died from hanging, contained double the quantity of gaseous contents of those who had died a natural death. This result, however, is certainly not correct; for there is always, as we have already stated, a very forcible expulsion of air from the lungs in the act of strangulation, and they are accordingly found almost empty after death. Mr. Coleman hanged an animal, and then secured the trachea by a ligature, and removed the lungs; when, upon receiving their gaseous contents in the hydro-pneumatic apparatus, he found their quantity was very far less than that which would have been collected under other circumstances.

III. *By Manual Strangulation.*—Whether strangulation be induced by the suspension of the body by the neck, or by a ligature drawn tight, or by any other pressure upon the trachea, the physiological phenomena of death are the same: where, however, the person has died from manual strangulation, the marks about the neck will probably be more evident, and the discoloration will correspond with the marks of the fingers and nails; and we may also expect to find traces of violence upon the chest, for, since the weight of the body is not obtained in such a case, additional force becomes necessary to consummate the fatal act. On opening the bodies of those who have been taken off by manual strangulation, Dr. Smith thinks that the usual appearances of this kind of death may not seem so conclusive as in other cases: an opinion in which we feel inclined to coincide; for, in consequence of the greater resistance of the sufferer, the functions of respiration and circulation may continue in some measure for a longer period than in drowning or hanging, which must be considered as more summary processes of suffocation. In the case of a woman who had been thus strangled by two men, Littre found the tympanum of the left ear lacerated, whence flowed about an ounce of blood; the vessels of the brain were unusually turgid, red blood was extravasated in the ventricles, as well as at the base of the cranium; the lungs were distended, and their membrane vascular. Not more, however, than an ounce of blood was found in the right ventricle of the heart, and it was fluid and frothy, like that in the lungs. This circumstance deserves particular notice, and can only be explained by supposing that the respiration and circulation were not at once arrested, but that the unhappy sufferer was enabled to inhale air, at intervals, during the protracted struggle. And yet, in certain cases, death may be very easily occasioned by manual strangulation; of which the murder of Dr. Clench, in the year 1692, may be adduced as an example: this gentleman was strangled in a hackney-coach by two men, while driving about the streets of the city, without the coachman having the slightest knowledge of the transaction, until he afterwards found him quite dead, kneeling down with his head on the seat, and a handkerchief bound about his neck, in which was a piece of coal, placed just over the windpipe.

IV. *By Smothering.*—In this act the transit of air into the lungs is prevented by forcibly closing the nostrils and mouth. It is very obvious that such a mode of destruction can very rarely occur in an adult; for a comparatively feeble resistance will be sufficient to overcome the assailant in such an attempt. It may, however, occur accidentally: it is not difficult to imagine that a person, in a fit of intoxication, may be unable to extricate himself from a position in which he might fall, and in which respiration could not be performed. In children, this mode of suffocation is less rare, and it may be either the result of design or accident; to which we shall have occasion to refer, when treating the subject of *Infanticide*.

V. *By the Inhalation of Air deprived of Oxygen.*—There are many gases, the inspiration of which occasions death: some of these act simply by excluding oxygen; while others exert an absolutely deleterious action, in consequence of the specific powers which they possess. It is exclusively to the first species that our attention is at present to be directed: the latter will constitute matter for future consideration, under the title of *Aërial Poisons*.

It is a fact too well established to require any discussion, that oxygen is the only principle which is capable of producing the necessary changes in the blood, during its transmission through the lungs; and that, accordingly, whenever atmospheric air is deprived of this principle, it is no longer capable of supporting life, and the animal immersed in it instantly dies. It is thus that death takes place from exposure to the fumes of charcoal, to those of lime-kilns, to the atmosphere of cellars, caverns, wells, and dungeons.

The asphyxia from privies, drains, and common sewers, depends upon a different cause, and will be considered under the head of *Sulphuretted Hydrogen*, in the history of Poisons.

The fatal effects of confined air in a small and crowded room, were fully exemplified in the year 1742, when twenty persons were crammed in a part of St. Martin's round-house, called the *hole*, during the night, several of whom died. The surgeons on that occasion gave it as their opinion, that, when the doors and windows were shut, the place could not support twenty persons for three hours without danger of their lives. A trial took place at the Old Bailey in consequence; but we have not been more successful than Dr. Gordon Smith in our search for its report. The medical jurist would be called upon, on such an occasion, for his opinion as to the nature of the deteriorated air, the causes of its accumulation, and whether it was adequate to the production of the alleged effects, and, possibly, whether the fatal consequences might not have been averted by judicious caution or active exertion. The most awful exemplification of the fatal effects of confined air is, however, recorded in the interesting narrative of what happened to the English in the *black hole* at Calcutta; and which we shall briefly relate in this place, as it involves some physiological phenomena, to which we shall hereafter have occasion to refer.

It was in the month of June, 1756, that the viceroy of Bengal laid siege to Fort William, the English factory at Calcutta. Mr. Holwell, assisted by the factors and the garrison, defended this post with extreme bravery, but was at length obliged to surrender. There were at this time remaining in the fort 145 men and one woman. The whole of this unfortunate company, many of whom were wounded, and several very dangerously, were shut up the same night in a small prison, only eighteen feet square. This prison, which is now better known in England by the name of the black hole, was enclosed by strong walls, and had only two small windows at one end, secured by iron grates. In this confined situation, which allowed only a space of about eighteen square inches to each individual, the heat and want of fresh air soon excited the most horrible effects: the prisoners, in a state of despair, began by attempting to force open the door, but in this they were unsuccessful. Mr. Holwell, who was placed near one of the windows, was more at his ease than the rest, and was consequently more cool and tranquil; and he recommended his companions to be quiet and orderly, and not to exhaust their strength by useless efforts. This advice produced some little calm, interrupted, however, by the groans of the wounded and the dying. The heat increased every moment. Mr. Holwell recommended them to strip off their clothes, as a means of acquiring more space: this was accordingly done, but with no great relief. They attempted to improve this by fanning the air with their hats; but even this was too painful a task for men who were worn out by the fatigue of the siege and the heat of this dungeon. Another of the company was for their kneeling down, that they might have more air. They all readily agreed to do this; and to rise together, in order to avoid confusion. This was done several times; but, every time the signal was given to rise, the number of those who had strength enough to obey it diminished. There were constantly some remaining on the floor, who were unable to get up, and these were trodden to death by the survivors. All this happened during the first hour of their imprisonment. At nine o'clock in the evening they began to complain of excessive thirst, and to renew their efforts to open the prison-door, and to tempt the centinels to fire upon them. Some of those who were farthest from the window became at once furiously delirious. The cry for water was unanimous. The guards brought water, and Holwell and two of his wounded friends received it at the window in their hats, and were going to pass it on to the rest; but, so eager and tumultuous were the efforts of the crowd to get at this water, that Holwell's two friends were suffocated, the water was spilt, and Holwell saw himself surrounded with dead bodies, who had either been crushed to death, or died for want of fresh air.

Hitherto the commander and benefactor of these unfortunate people had been treated with some degree of respect, but now all distinction began to be forgotten: the whole company eagerly threw themselves towards the windows, and, seizing the iron bars, some of them got even upon his shoulders. He was so borne down by this enormous weight, as to be deprived of all power of motion: he implored the pity of those who were upon his head and his shoulders, and requested them to let him go and die at the bottom of the prison. This request was readily complied with; every one was desirous of succeeding to his place, and without much difficulty he reached the farther end of the dungeon. The third part of these unhappy people were already dead; and they who were still alive pressed so eagerly towards the windows, that Holwell found himself somewhat freer in his new station; but the air was so corrupted, that his breathing soon became extremely difficult and painful. Unable, therefore, to support this, he attempted once more to make his way to the windows; and, leaning on a heap of dead bodies, he now resolved to wait patiently for death. In this situation he remained about ten minutes, and then he experienced such a pain of the breast, and so violent a palpitation of the heart, that he was obliged to make one more attempt towards getting a less fatal air. There were five rows of his companions between himself and the window; his despair carried him through four of these. The palpitation of his heart now began to abate, but he felt inexpressible thirst, and cried out for water; but the water seemed to increase, instead of alleviating, his thirst: he therefore resolved to drink no more, and rather chose to suck the moisture from his shirt, which seemed to afford him some relief. A young man, quite naked, who stood before him, eagerly seized the sleeve of his shirt, and for some moments deprived him of this salutary refreshment. It was not yet midnight. The small number of those who were left were transported to the greatest excess of rage and despair. They all called aloud for air, because the water that had been brought to them afforded no relief. Soon after this the noise suddenly ceased. The greater part who were living laid themselves down, deprived of all their strength, and peaceably breathed their last. Others aimed at getting into Holwell's situation; a Dutchman mounted on one of his shoulders, and a black soldier on the other. In this situation he remained till two in the morning, when he gave up his place to a marine officer, who was soon forced out of it by the Dutchman. The officer retired with Holwell to the other corner of the prison, and in a few moments afterwards died. Holwell himself was soon deprived of sense, and from that time till sun-rise we have no account of what passed. One of those who remained alive at five in the morning, drew forth Holwell from the heap of dead, and found in him some signs of life. About that time the viceroy inquired whether he was still alive? he was told that, if the door was immediately opened, it would perhaps be possible to recover him, and orders were accordingly given for this purpose. But the door of the prison opened inwards, and they who were within it, and living, were deprived of all their strength, so that more than twenty minutes elapsed before the dead bodies were removed, which prevented the door from being opened.

At a quarter after six o'clock, there came out of this melancholy dungeon three and twenty persons, the remains of the hundred and forty-six who had entered it on the preceding evening.

Upon the events thus related we have to remark, that no advice could be more judicious than that given by Holwell to his companions in the early part of their imprisonment, "to be quiet and orderly, and not to exhaust their strength by useless efforts." Nor can we imagine any measure more calculated to increase the sufferings of their situation than that which was subsequently proposed, and adopted, by another of the company, "to fan the air with their hats, and to kneel down and rise together, by a simultaneous motion." It has been satisfactorily established by physiological researches, that the demand for oxygen, in an animal body, will be in proportion to its expenditure by muscular exertions: whenever, therefore, circumstances may render a supply of air deficient, we shall best economise that which we possess by perfect quiet. Lavoisier says, that a man, under ordinary circumstances, consumes 1300 or 1400 cubic inches of oxygen in an hour; but he found that, if he is engaged in raising weights, the consumption is at the rate of 3200 in the hour.

Infants appear to be less able to sustain the deprivation of oxygen than adults; and, in some cases on record, life has been destroyed by circumstances that we should have, *a priori*, considered as hardly adequate to such an effect. A case is related of a child, who was suffocated by some drunken men having repeatedly blown out a candle, and held the smoking wick under its nose. The faculty of Leipsic investigated the circumstances, and declared the death to have taken place in consequence of suffocation. (*Valentini Pand. Med. Legal.* sect. ii.)

VI. *By other Modes, not included in the foregoing Sections.*—We have already stated that, if the muscles of respiration be paralysed, the animal can no longer breathe, and it dies in a state of suffocation. There are several mechanical modes by which such a condition may be produced: a person buried in a heap of ruins, although his head should be free, will perish from the pressure of the surrounding rubbish preventing the due action of the respiratory muscles. It was in this way that criminals, who obstinately refused to plead, often died under the pressure of the weights that were heaped upon their bodies.

There is a mode of suffocation described by Galen, as being practised by the slaves, when brought into the presence of the judges or executioners: it consisted in swallowing their tongue, by which it is said they voluntarily terminated their own existence. Several more modern authors have noticed this incredible mode of suicide, as one that is resorted to by negroes. Now, to confute such an idea, we have only to show the attachment of the muscles of this part, and the motions which they permit. Equally absurd is it to suppose, with other physiologists, that persons can occasion suffocation by a voluntary suspension of their breathing; for, if such an attempt were even made, the effort would be ended when self-possession was once lost, for then the impulse of nature must instantly triumph over any struggle to oppose it. We are not, however, prepared to say, that such an attempt might not, in certain cases, occasion such a cerebral congestion as to produce apoplexy.

The last cause of suffocation which we have to mention is mechanical obstruction, from the entrance of foreign bodies into the aperture of the glottis. Instances of this kind are too numerous and familiar to require many observations: it is thus that Anacreon is said to have perished from a grape-seed. Gilbert, the poet, terminated his existence in a similar manner: he was a man of great appetite, and, in the midst of a festival, went into a neighbouring room, but did not return, to the great surprise of his convivial companions. He was found stretched on a couch, without any signs of life. The assistance administered by his kind, but uninformed, friends was useless. On opening the body, a small piece of mutton was found, that had stopped at the entrance of the larynx, and completely prevented the passage of air into this organ. In October, 1821, two inquisitions were taken at Mildenhall, before the coroner of Bury St. Edmonds, in Suffolk: in the one case it appeared that John Harris had eaten some honey from the honey-comb, and that a bee, having been concealed in it, entered the glottis, and occasioned almost immediate death by suffocation; the other case was that of an infant, Mary Bacon, who fell with her face upon a quantity of slacked lime, when a particle of it getting into the windpipe, produced inflammation of the lungs and sloughing of the trachea, of which she died. We have no doubt but that persons, during the state of intoxication, or that of a spasmodic paroxysm, have often perished from suffocation, when the death has been attributed to other causes. If the stomach should reject its contents during a state of insensibility, such an occurrence is by no means unlikely. We have lately received the history of a case of this description, which occurred in the St. James's workhouse, and fell under the particular notice of Mr. Alcock. The patient was seized, after a hearty meal of pork, with an epileptic fit, during which he died; when, upon opening the trachea, it was found to contain a quantity of animal matter, resembling the pork upon which he had recently dined.

---

